# Effects of binocular cue availability on leaping performance in *Cheirogaleus medius*: implications for primate origins

**DOI:** 10.1242/jeb.245434

**Published:** 2024-02-22

**Authors:** Addison D. Kemp

**Affiliations:** Department of Integrative Anatomical Sciences, Keck School of Medicine, University of Southern California, Los Angeles, CA 90033, USA

**Keywords:** Grasp-leaping hypothesis, Locomotion, Arboreal habitat, Visual adaptations, Binocular field

## Abstract

Multiple competing hypotheses attribute the evolution of the suite of traits that distinguish primates from their closest relatives, including forward-facing eyes, which create a wide field of binocular vision, to specific behavioral and ecological factors. The grasp-leaping hypothesis suggests that the evolution of these traits in basal primates was driven by the demands of a form of leaping locomotion unique to primates. Whether the grasp-leaping hypothesis provides a viable mechanism for the evolution of primates' forward-facing eyes remains untested. To determine whether grasp-leaping locomotion may have contributed to driving the evolution of primates' forward-facing eyes, the importance of vision within the binocular field for this type of leaping was evaluated experimentally in *Cheirogaleus medius*, one of the cheirogaleid primate species considered reasonable living analogs of the earliest primates. Availability of binocular visual cues was experimentally restricted using a head-mounted blinder that narrowed the binocular visual field without altering the total visual field. Animals altered their launch behavior, reduced their horizontal leap speed, and were significantly more likely to select paths that offered the shortest available leaps when their binocular field was restricted. Restriction of binocular cue availability also significantly increased the probability of adverse landings even when statistically controlling for potentially confounding variables such as leap distance, horizontal leap speed, learning effects, etc. These results suggest a functional mechanism by which selection for improved grasp-leaping could also have contributed to the evolution of forward-facing eyes in the earliest crown primates.

## Teaser

**Highlighted Article:** Cheirogaleid primates with restricted binocular visual cue availability alter their leaping behavior and experience more frequent adverse landings, suggesting the evolution of forward-facing eyes potentially facilitated leaping in early primates.

## INTRODUCTION

Forward-facing eyes with convergent optic axes are one of the most distinctive features of the primate order and differentiate living primates from most other living mammals. There is also a stark contrast between the convergent bony orbits of fossil crown primates and the laterally directed orbits of primates' closest living relatives (tree shrews and colugos), and crown primates' closest fossil relatives (Plesiadapiformes) (Kirk et al., 2003; Bloch et al., 2007). While orbital convergence, or the degree to which the bony orbits face the same direction (Cartmill, 1974), is often exceeded by optic axis convergence in mammals, there is still a strongly predictive relationship between the two variables (Kemp, 2019), meaning these differences in orbit orientation also support forward-facing eyes as a derived feature unique to crown primates among their close relatives (Kirk et al., 2003; Ni et al., 2004; Ravosa and Savakova, 2004; Ross et al., 2007). New fossil material increasingly suggests that other features originally used to diagnose living primates as a clade relative to other living mammals (e.g. a divergent hallux, relatively long manual digits, bunodont molars) may have evolved in a mosaic fashion in the primate stem lineage (Bloch and Boyer, 2002; Bloch and Silcox, 2006; Bloch et al., 2007; Kirk et al., 2008; Boyer et al., 2013a, 2016; Silcox et al., 2014). Nevertheless, forward-facing eyes remain a characteristic associated exclusively with the crown primate clade among the Euarchonta (primates, tree shrews and colugos). Despite the importance of forward-facing eyes as a trait associated with the origin of crown primates, significant debate continues over the adaptive significance of this feature. Several adaptive scenarios have been put forward to explain the evolution of diagnostic primate traits, including forward-facing eyes. While two adaptive scenarios focus largely on pressures exerted by foraging demands (Cartmill, 1972, 1992; Sussman and Raven, 1978; Sussman, 1995; Sussman et al., 2013), the grasp-leaping hypothesis suggests that early primate adaptations were driven by a type of leaping locomotion in which small-bodied animals navigated their arboreal habitat by launching from one support and landing on the next substrate with a very precise grasp (Szalay and Dagosto, 1980, 1988; Szalay, 2007).

However, functional morphological analyses of the ankles of stem and crown primates as well as ancestral reconstructions of primate foot and ankle morphology suggest that the earliest crown primates would not have been as adept at or as specialized for leaping as extant primate leaping specialists such as tarsiers and some small galagos (Boyer et al., 2013a; Marigó et al., 2016). Instead, there appears to have been parallel adaptation for increased leaping specialization within both the strepsirrhine and haplorhine lineages (Boyer et al., 2013a; Gebo et al., 2012, 2015; Marigó et al., 2016). The earliest primates and their immediate ancestors are reconstructed as having leaping abilities more similar to those of extant small-bodied generalized arboreal quadrupedal primates, including *Cheirogaleus* (Boyer et al., 2013b).

Selection to improve leaping locomotor efficiency provides a logical explanation for the evolution of several distinctive features of the limbs (Boyer et al., 2013a), in the earliest crown primates. However, the functional mechanism by which selection for improved leaping efficiency could lead to the evolution of forward-facing eyes is less intuitive. The evolution of more forward-facing eyes increases an animal's binocular field at the cost of reducing its total panoramic visual field. However, there are several benefits to this eye orientation (reviewed in Read, 2021). Light within the binocular field has an up to 2× greater chance of being captured, improving light sensitivity in this region (Pirenne, 1943; Warrant, 2008), which would be advantageous for nocturnal animals such as the earliest crown primates. Forward-facing eyes with more parallel optic axes also increase the acuity of an animal's vision in the region in front of their snout by reducing spherical aberration of light passing through the lens from this region (Pettigrew, 1986). Again, this is an important advantage in nocturnal species living in light limited environments that preclude the use of pupil constriction to counter spherical aberration. Depth perception within the binocular field is more precise than within the monocular visual fields as a result of two depth perception mechanisms that require visual input from two eyes – vergence and stereopsis (Poggio and Poggio, 1984; Howard and Rogers, 1995; Plooy et al., 1998; Purves et al., 2012). Contrast discrimination, the ability to detect luminance discrepancies between objects or among regions within an object, is also improved within the binocular field (Pirenne, 1943; Campbell and Green, 1965). Optic flow provides information to a moving animal about the direction and speed of its own movement via the impression that objects in the visual scene that are closer to the individual and further from the direction of travel are moving faster (Lee, 1980; Lee and Reddish, 1988; Gray and Regan, 1998; Warren et al., 2001). Forward-facing eyes with a wide binocular overlap position the center of expansion, a point aligned with the direction of travel that does not move within the visual scene as an animal moves forward, closer to the central retina where primate photoreceptor densities are higher, which may improve the precision of optic flow cues.

Studies of living primates inferred to be similar to the earliest crown primates in their use of leaping, their nocturnality and their small body size have suggested that acrobatic arboreal leaping may be dependent on accurate depth perception (Martin, 1979; Crompton, 1995). A wider potential field of more accurate depth perception offered by more forward-facing eyes may allow the animals to more accurately gauge the distance to their landing substrate. However, it has been suggested that stereopsis may not function over the distances crossed during potential grasp-leaping in the earliest crown primates (Heesy, 2009). It is also possible that either a narrow binocular field or the numerous depth cues available through the entire visual field (including motion parallax, perspective cues, etc.) could have been sufficient to facilitate the type of leaping behavior used by the earliest crown primates and their immediate ancestors (Rock, 1984; Kandel, 1991; Heesy, 2009). Even if either of these scenarios regarding depth cue importance for leaping and ancestral primates is correct, the increased light sensitivity, increased contrast discrimination, improved acuity and potential optical flow improvements afforded within the binocular field by forward-facing eyes may also have played a potentially important role in facilitating leaping behavior in a nocturnal arboreal crown primate ancestor.

Here, an experimental approach was used to evaluate the effect of binocular cue availability on grasp-leaping locomotion in a small-bodied, generalized arboreal quadrupedal primate. Leaping performance was quantified in captive *Cheirogaleus medius*, a species inferred to be similar to the earliest primates in the extent of their leaping adaptations (Boyer et al., 2013a), both under normal visual conditions and with the width of the binocular field reduced using a medial blinder. The results of this work provide the opportunity to determine whether having a wider binocular field confers a performance advantage in leaping and therefore whether there is a viable functional mechanism by which the demands of grasp-leaping type behavior could have contributed to selection for forward-facing eyes in primates.

## MATERIALS AND METHODS

### Sample

Subjects consisted of three male (individuals A–C) and two female (individuals D and E) fat-tailed dwarf lemurs (*Cheirogaleus medius* É. Geoffroy 1812) born and raised at the Duke Lemur Center, USA. All individuals were adults whose recent ophthalmological exams indicated they were free of any visual pathologies (e.g. cataracts, etc.). Body mass in *C. medius* fluctuates throughout the year as a result of seasonal hibernation but there is no significant body mass dimorphism in this species. All experimental sessions were conducted at least 2 months before the onset of torpor and before the subjects' body mass changed substantially. All training and experiments were completed at the Duke Lemur Center under Duke University IACUC protocol A062-16-03 and University of Texas IACUC protocol AUP-2017-00041. To prioritize animal welfare, training was based exclusively on positive reinforcement, the use of short-term manual restraint was limited, and the experimental enclosure was designed to minimize required handler/animal interaction. All training and experimental procedures were categorized as non-painful by the IACUC.

**Fig. 1.**
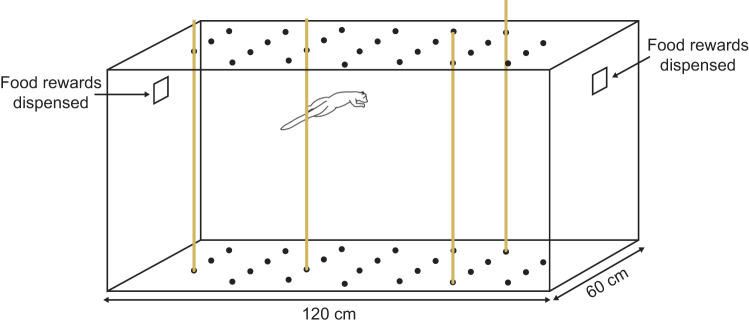
**Diagram of the experimental enclosure with subject leaping.** Grid of potential vertical substrate positions visible on the floor and ceiling of the enclosure.

Subjects were trained to voluntarily leap between narrow (1 cm diameter) vertical substrates to cross a 60×60×120 cm enclosure (Fig. 1) in exchange for grape slices that were offered through openings near the ceiling at each end of the enclosure. The enclosure had a grid of 26 positions into which the vertical supports could be placed (Fig. 1). During training as well as trials, rewards were only offered to animals when they crossed the enclosure by leaping between supports to discourage them from walking along the floor of the enclosure. Crossing the floor of the enclosure was rare and animals learned the task quickly, requiring only two training sessions each before voluntarily leaping >75 times per session. Positions of the vertical supports were randomized throughout the course of each experimental session, which allowed for data on leaps of varying distances to be collected for a single subject within a single session. An average of 63 leaps elapsed between pole changes. Randomizing the substrate positions allowed the extent to which subjects could rely on memory from past leaps to gauge distance between substrates to be restricted. The randomization also allowed for such potential learning effects to be accounted for explicitly in models (below). Substrate positions were changed following a list of position combinations that randomized the number of substrates and their positions within the constraints that all combinations had minimum possible leap distances of no less than 10 cm and no more than 45 cm. These constraints ensured that the subjects, roughly 15 cm from shoulder to hip, could always cross the enclosure but did not rely extensively on bridging behavior (contacting the landing substrate before removing their feet from the starting substrate).

**Fig. 2.**
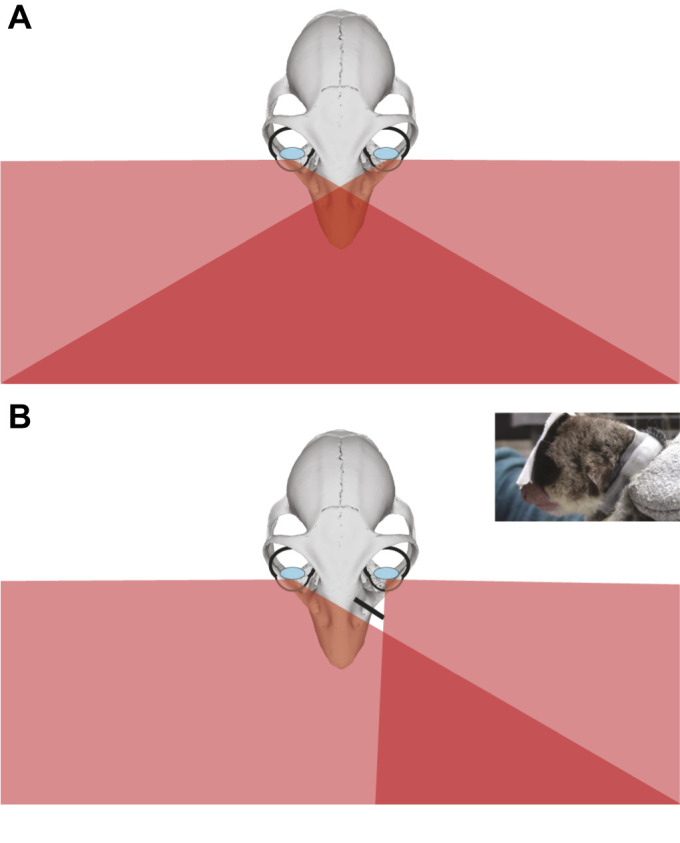
**The restricted binocular field condition.** (A) Schematic diagram of the visual fields of *Cheirogaleus medius* based on the estimated binocular field of other strepsirrhine species with similar degrees of orbital convergence (Ross, 2000; Heesy, 2004). (B) Schematic diagram of binocular field reduction (see inset) achieved by the medial blinder (represented by the black bar). *Cheirogaleus medius* skull adapted from a surface model downloaded from morphosource.org (Cheirogaleus_major_USNM100640_cranium.ply, AMNH specimen 100640).

Each subject participated in four experimental sessions, two under a control condition and two under a restricted binocular field condition. The restricted binocular field condition was produced using helmets with 1 cm obliquely angled medial blinders which substantially restricted the medial visual field of the left eye (Fig. 2). The extent of occlusion was measured by fully covering the eye not occluded by the medial blinder and passing an object across the animal's visual field, moving from the fully covered side to the partially occluded side, 10 cm in front of the animal's snout. This was done until the animal responded to the stimulus, indicating the medial margin of the visual field of the eye occluded with the blinder. The blinders restricted the medial visual field of the occluded eye by ∼45 deg. All subjects tolerated the helmets well, anesthesia was not required to fit the helmets at the start of the sessions and manual restraint for placing the helmets was roughly 1 min. The helmet was repositioned no more than twice during a session if the subject managed to knock it out of place. If the helmet was knocked out of place more than twice, the session was ended. For the control sessions, the subjects wore similar helmets that lacked a medial blinder, allowing their full range of binocular vision but making any distraction or performance deficit attributable to the helmet itself consistent across all trials.

All sessions were filmed at 115 frames s^−1^ using a Baumer VCXU-23M camera with a Sony IMX174 sensor and an Edmund Optical 25 mm lens. The lighting conditions in the experimental room were the same as the conditions used in the subject's home enclosures during their waking period to mimic the subject's natural nocturnal habitat. Infrared ring lights (Siemens) producing wavelengths detectable to the cameras but not visible to the lemurs were used to provide sufficient light to allow recording at this frame rate. The red lights used in the subject's housing enclosures during their active periods were also used during the sessions. Pixel grayscale values were binned in both the *x*- and *y*-axes to increase the brightness and contrast of the resulting video. A total of 2592 leaps were recorded, 1492 under the control condition (mean±s.d. 298.4±63.1 per animal) and 1100 under the reduced binocular field condition (220±40.5 per animal).

The first frame in which no appendages were in contact with the substrate was recorded as take-off. In the few cases where this guideline could not be used because of visibility limitations (<2% of trials), take-off was recorded as the first frame in which the substrate showed recoil at the start of a leap. The first frame in which any part of the subject's body contacted the landing substrate was recorded as the landing frame.

### Performance variables

All performance variables were scored or calculated based on a review of the leap recordings. Performance variable operational definitions were refined using a separate pilot dataset of 200 leaps collected in June 2016.

#### Landings

Landings were scored as one of five types (see Movies 1–4). In a spin (Movie 1), the subject is not able to counter the rotational momentum it creates upon landing and rotates at least 90 deg around the pole. In a strike (Movies 2 and 3), the subject's face or torso contacts the landing substrate before their hands or feet. In a drop (Movie 3), the subject fails to get a secure grip on the substrate and falls at least 5 cm before managing to arrest themselves. In a fall (Movie 4), the subject completely fails to contact the vertical substrate and lands on the bottom of the enclosure. A normal landing was any landing to which none of these descriptions applied. All non-normal landing types were categorized as adverse landings. While a fall is obviously detrimental, spins, drops and strikes are all indicative of a failure of the animal to prepare adequately for landing. These errors will require additional compensatory movements upon landing and preclude the type of rapid, precise, serial leaping the grasp-leaping hypothesis invokes. In the small number of cases where a landing exhibited both a spin and a drop or a strike and a drop, the landings were recorded as drops. Additionally, drops and falls, both of which may potentially incur significant fitness detriments in the wild, were categorized as potentially catastrophic landings.

#### Distance

The leap distance was calculated as the minimum horizontal distance between the starting and ending substrates for a leap. Changes in vertical position were not included in this calculation.

#### Speed

Horizontal leap speed was calculated as [distance between substrates (m)/landing time−take-off time (s)]. The oblique path taken across the enclosure in the majority of leaps precluded measurement of take-off and landing velocities as space limitations restricted filming to a single camera set up perpendicular to the long side of the experimental enclosure. This measure is not comparable to measures of take-off or landing velocity as it (1) does not take into account any vertical distance traveled, and (2) is based on the relative timing of hindlimb lift-off at take-off and forelimb touchdown at landing, rather than tracking the movement of a single point on the animal (e.g. center of mass, eye) over time. This variable was modeled as an outcome variable to determine whether binocular field restriction caused subjects to adjust the mechanics of their leap. Speed was also included as a potential predictor variable in models of other performance outcome variables to account for the possibility that the horizontal speed of a leap, regardless of visual condition, may also have an impact on landing type, etc.

#### Motion parallax

If a subject exhibited a repetitive head movement perpendicular to the direction of the leap while visually orienting toward the landing substrate before taking off, the use of motion parallax was recorded for that leap.

#### Adjustments

The number of grasp adjustments was recorded as the number of times the subject regripped the substrate with any appendage between a landing and the subsequent take-off. If the subject climbed the substrate before initiating another leap, climbing steps were not counted as grasp adjustments. A climbing step was defined as the replacement of an appendage on the substrate in which the appendage was translated along the substrate in the same direction as the movement of the subject's body (this movement had to last for at least two steps) rather than a replacement that only changed the appendage's grasp on the substrate or position relative to the other appendages without significant translation in the direction of subject movement.

#### Path

Whether the subject chose the shortest available leap was determined automatically using a custom R script which compared the distance from the starting pole to the landing pole with the distance between the starting pole and each alternative landing substrate.

#### Focus

This variable recorded the first frame in which the subject oriented its head toward the landing substrate before taking off. This frame was identified by reviewing frames in reverse order starting with the take-off frame and progressing until the subject aligned its head away from the landing substrate. Precision and reliability of this variable were evaluated by repeat scoring in a subset of 50 launches. Focus frame values varied by an average of 1.2±0.4 frames or 10.4±3.5 ms in repeat scorings, indicating considerable reliability.

#### Take-off lag

Take-off lag was calculated as the time between when the subject focuses on a landing substrate and when it takes-off (i.e. take-off frame−focus frame).

### Statistical analyses

Each performance variable was modeled separately using a generalized linear mixed effects model (GLMM) with ‘subject’ as a random variable. Binary performance variables (whether a landing was adverse, whether a landing was catastrophic, whether motion parallax was used) were modeled using a binomial distribution using a logit link function. A nominal categorical variable including all landing types was modeled using a multinomial logistic regression with a logit link function. Take-off lag was binned into 20, 250 ms bins, and count variables (number of adjustments, take-off lag) were modeled using a Poisson distribution. Appropriateness of Poisson distributions was evaluated through chi-squared tests (*P*>0.05 in all cases). Distance, take-off lag and speed were all log_10_ transformed. The continuous response variable (speed) was not normally distributed despite log_10_-transformation, but was still included as a response variable given the roughly symmetrical distribution of the errors and the large sample size, and as well as the robusticity of linear mixed effects models to violations of the normality assumption (Schielzeth et al., 2020). The predictor variables leap and leap since pole change were rescaled and centered to avoid high eigenvalues in GLMMs (Moore and McCabe, 1999).

Best-fit models for each performance variable were identified through a backward elimination process. All potentially relevant independent variables were included in the initial model and subsequent models were run after eliminating the independent variable with the highest *P*-value. Potential learning effects were accounted for by including the number of leaps since the last pole position randomization in all initial models. This process was stopped when all independent variables in a model had a significant effect on the performance variable. Interaction terms were omitted from the models because there were no *a priori* expectations of interactions between independent variables. Bayesian information criterion (BIC) values were compared between models to identify the best-fit model. The BIC was selected because it prioritizes model parsimony by more heavily penalizing model complexity (Schwarz, 1978). Results reported here are based on the best-fit models for all performance variables. All models run, along with their BIC values, are included in Table 1. To complete a *post hoc* evaluation of the potential interaction between condition and leap distance, this term was added to the previously identified best-fit models and BIC was evaluated. This led to improved model fit for one outcome variable (speed). An alpha value of 0.05 was used for all models other than the two models that used landing type as a dependent variable (adverse landings and catastrophic landings). For those two models, a Bonferroni correction was applied, and the alpha value was thus set to 0.025. All data processing and analyses were completed using the R statistical environment (http://www.R-project.org/) and the *dplyr* (https://CRAN.R-project.org/package=dplyr), *lme4* (Bates et al., 2015), *lsmeans* (Lenth, 2016) and *ggplot2* (Wickham, 2009) packages.

**Table 1.**
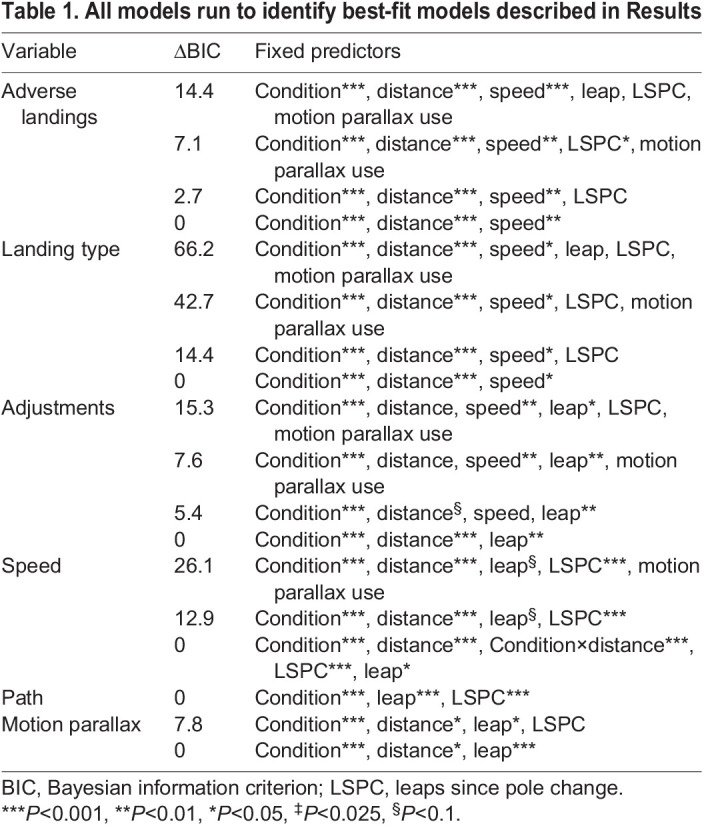
All models run to identify best-fit models described in Results

## RESULTS

### Landings

**Fig. 3.**
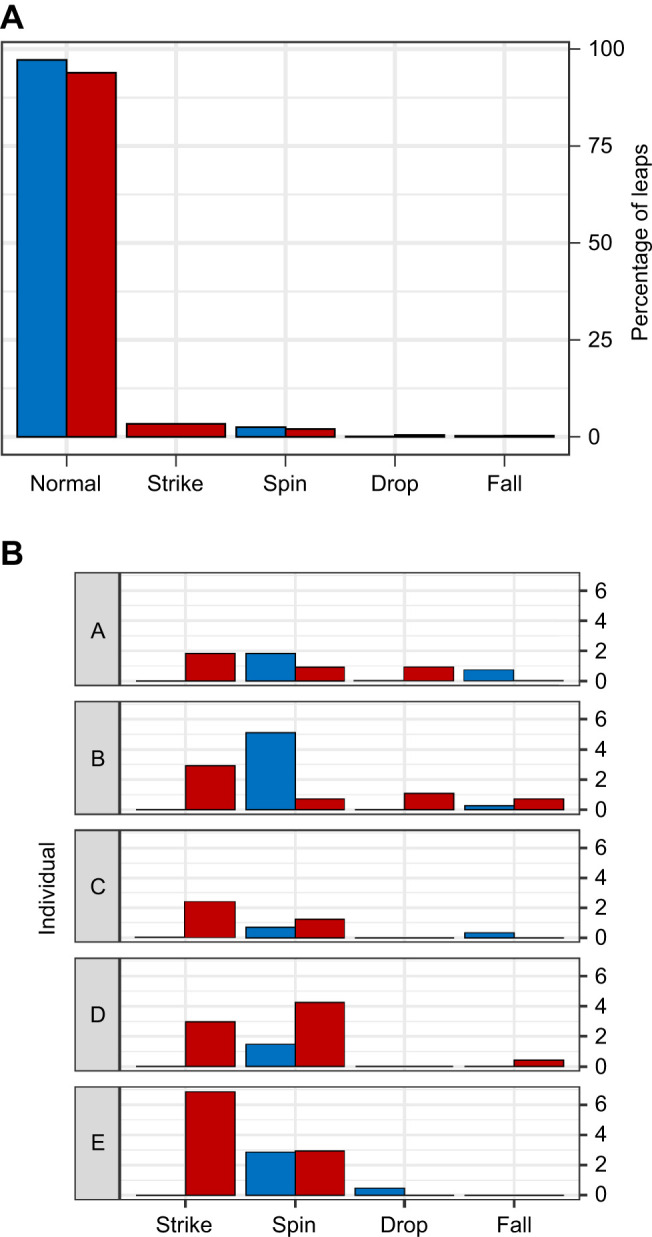
**Effect of binocular cue availability on landing types.** (A) Proportion of leaps resulting in each landing type (*n*=2592 leaps pooled across all five individuals). Binocular field restriction (red) significantly increased the probability of adverse landings (*P*<0.001) compared with control (blue) in the best-fit model when controlling for the significant effects of distance (*P*<0.001) and speed (*P*<0.01). (B) Proportion of leaps that resulted in each type of adverse landing plotted for each individual (A–E).

Having a restricted binocular field made subjects significantly more likely to experience an adverse landing (binomial GLMM, *P*<0.001, *z*=6.31; Fig. 3). Controlling for the effects of other variables, subjects had a 6.4±1.1% probability of having an adverse landing while wearing a blinder compared with a 1.3±0.3% probability under the control condition. Longer leap distances and slower horizontal leap speeds also significantly increased the likelihood that a subject would have an adverse landing (binomial GLMM, both *P*<0.001, *z*_distance_=4.286, *z*_speed_=−4.341). This increase in the probability of adverse landings under the restricted binocular field condition was driven by a dramatic increase specifically in strike landings under that condition (multinomial GLMM, *P*<0.001, *z*=13.929). None of the potential explanatory variables, including restriction of the binocular field, had a significant impact on the probability that a subject would have a potentially catastrophic landing – a drop or fall (binomial GLMM, *P*=0.29, *z*=0.014; Fig. 3).

### Other parameters

**Fig. 4.**
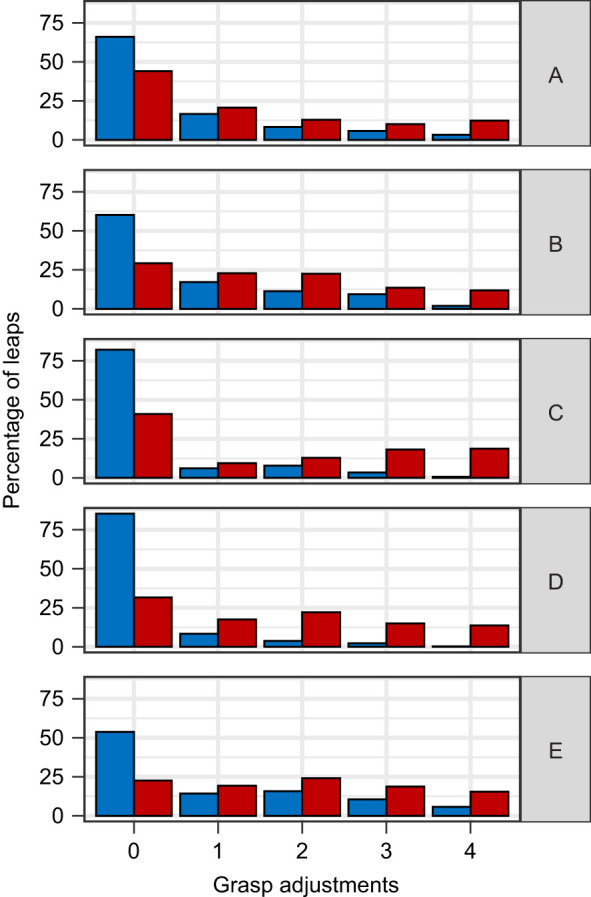
**Number of grasp adjustments required during leaps under control and reduced binocular field conditions for each individual subject (A–E) across all 2592 leaps.** Binocular field restriction (red) significantly increased the number of grasp adjustments made after landing (*P*<0.001) compared with control (blue) in the best-fit model when controlling for the significant effects of distance (*P*<0.001) and leap number (*P*<0.05).

**Fig. 5.**
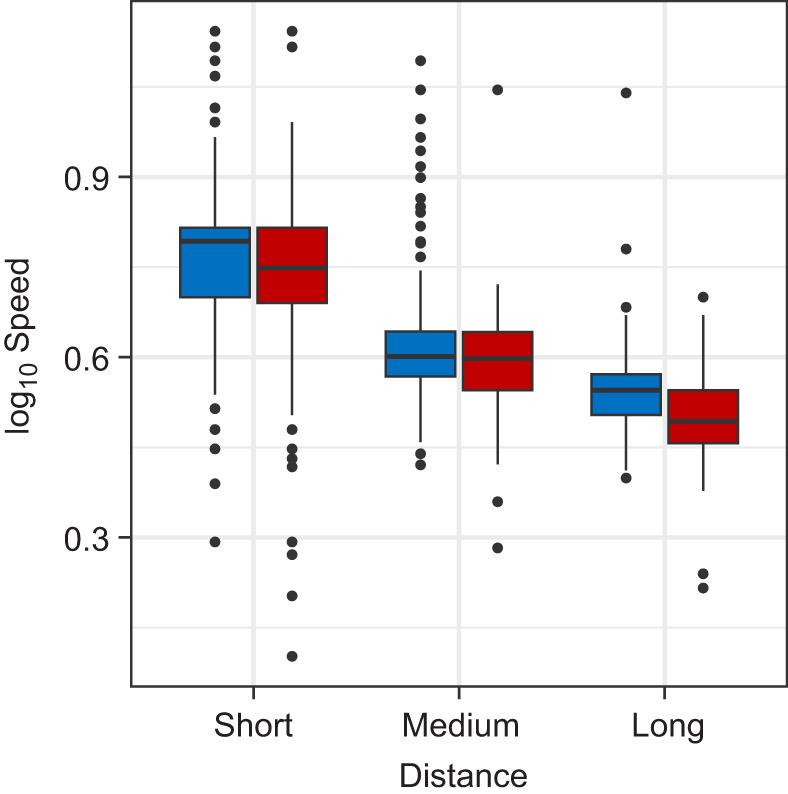
**Effect of binocular field restriction on horizontal leap speed.** Log_10_ horizontal leap speed (m s^−1^) under control (blue) and reduced binocular field (red) visual conditions for short (<35 cm), medium (35–60 cm) and long (>60 cm) leaps (box plots show median, upper and lower quartiles and 1.5× interquartile range). Binocular field restriction significantly decreased horizontal speed (*P*<0.001) in the best-fit model, which also accounts for the significant effects of distance (*P*<0.001), an interaction between distance and condition (*P*<0.001), leap number (*P*<0.01), and the number of leaps since the last pole randomization (*P*<0.001).

**Fig. 6.**
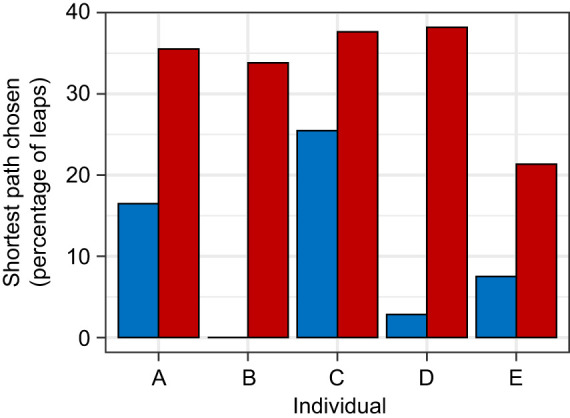
**Frequency with which the five study subjects (A–E) selected the shortest available path under control and reduced binocular field conditions.** Binocular field restriction (red) significantly increased the probability that an animal would select the shortest available leap (*P*<0.001) compared with control (blue) in the best-fit model, which also accounted for the significant effects of leap number (*P*<0.001) and number of leaps since the last pole randomization (*P*<0.001).

**Fig. 7.**
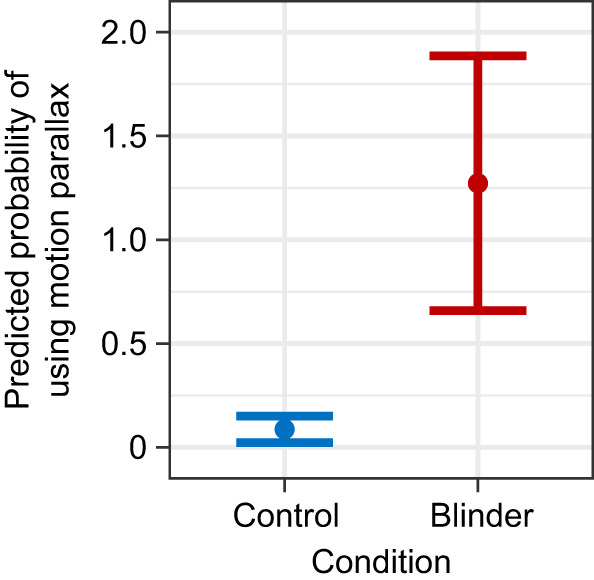
**Predicted probability that a subject would use motion parallax under control and reduced binocular field conditions.** Probabilities are shown for the control (blue) and binocular field restriction conditions (red), controlling for all other significant predictors of motion parallax use. Error bars are confidence intervals for the probabilities based on the best-fit motion parallax model, which also included significant effects of distance (*P*<0.001) and leap number (*P*<0.01).

Having a restricted binocular field caused the subjects to make significantly more grasp adjustments between landing and initiating another jump (GLMM, *P*<0.001, *z*=16.706; Fig. 4). The number of adjustments also increased on longer distance leaps (GLMM, *P*<0.001, *z*=3.911) and decreased as the number of previous leaps the subject had completed increased (GLMM, *P*=0.004, *z*=−2.845). Controlling for the effects of other variables, subjects averaged 1.6 adjustments per leap under the control condition and 2.5 adjustments under the restricted binocular field condition. Subjects leapt at significantly slower horizontal speeds while under the restricted binocular field condition (GLMM, *P*<0.001, *z*=46.558, Fig. 5), as well as when leaping longer distances (GLMM, *P*<0.001, *z*=−3.550; Fig. 5). The number of leaps the subject had already completed that session and the number of leaps the subject had completed since the last substrate randomization also significantly increased horizontal leap speed (GLMM, *P*_leap_=0.029, *z*_leap_=2.191, *P*_LSPC_<0.001, *z*_LSPC_=−6.266, where LSPC is leaps since pole change). Having a restricted binocular field also made the subjects significantly more likely to choose the shortest path available (binomial GLMM, *P*<0.001, *z*=12.690; Fig. 6). Animals were also more likely to choose a shorter path earlier in the trial session (binomial GLMM, *P*<0.001, *z*=−4.863) as well as soon after reconfiguration of the substrates (binomial GLMM, *P*<0.001, *z*=4.025). After statistically controlling for these potential learning effects, subjects had a 7.2±1.2% chance of choosing the shortest path under the control condition versus a 26.7±3.5% under the restricted binocular condition. Subjects were significantly more likely to use motion parallax when they had a restricted binocular field (binomial GLMM, *P*<0.001, *z*=4.052, Fig. 7), as well as when they were leaping longer distances (binomial GLMM, *P*=0.013, *z*=2.494) and earlier on in a trial session (binomial GLMM, *P*<0.001, *z*=−3.334). Take-off lag was highly variable under both visual conditions and neither binocular field restriction nor any other potential predictor had a significant effect on take-off lag.

## DISCUSSION

When subjects leapt with a restricted binocular field, they chose to cross shorter distances, leapt more slowly, were more likely to have adverse landings, were more likely to attempt to use a monocular depth cue, and needed to make more adjustments to their grasp on the substrate before taking off again. While the subjects were not more likely to experience a potentially catastrophic outcome when they had a restricted binocular field, the above changes in performance and behavior all suggest that the subjects struggled more with distance estimation when their binocular field was restricted. Increases in adverse landings and grasp adjustments reveal that leaps and landings were less precise when subjects were not able to use their full binocular field. Subjects also moved more conservatively in terms of both horizontal speed and path choice under the restricted binocular field condition. While the specific differences observed between blinder and control conditions suggest the impacts of the blinder are mostly attributable to the disruption of the binocular field, it is possible that the presence of the blinder in one visual field but not the other may have created a rivalrous precept that may have also impacted the observed results.

Two aspects of the landing type results suggest that subjects are adjusting their leaping approach to accommodate uncertainty about substrate distance when leaping with a reduced binocular field. First, the most common adverse landing under the restricted binocular field condition was the strike, in which a subject fails to raise their hands in preparation for landing and therefore hits the substrate with their face or chest. This landing type never occurred under the control condition. The dramatic increase in strikes, which occur because the subject fails to prepare for landing in time, under the reduced binocular field condition suggest that subjects are overestimating leap distances when their binocular field is restricted. Second, under both visual conditions, all leaps that ended with a subject missing the substrate entirely were always due to improper aim rather than the subject failing to propel itself far enough to reach the next substrate. The frequency of adverse landings in which subjects failed to prepare for landing quickly enough (e.g. strike) and the fact that subjects never ‘shorted’ a leap both suggest that the subjects are erring on the side of overestimating leap distance when faced with uncertainty about distance under the reduced binocular field condition. While overestimating leap distances leads to subjects forcefully contacting the substrate with their snout or trunk, this adverse landing is preferable to a potentially lethal fall from the trees that may result from underestimating leap distances.

Reliance on monocular depth cues could only be determined behaviorally for one cue – motion parallax. Subjects were significantly more likely to use motion parallax before take-off to gauge distance to the landing substrate when the availability of binocular cues was restricted; however, this was still an uncommon behavior under both visual conditions (occurring in 0.2% of control leaps and 2% of blinder leaps). The increased use of motion parallax under the reduced binocular cue condition suggests that subjects were seeking alternative cues to substrate distance when the availability of binocular cues was restricted. This result also suggests that subjects experienced a reduced ability to estimate distance when their binocular field was narrowed. If depth perception based on motion parallax was sufficient to facilitate grasp-leaping behavior, it would be expected to significantly decrease the probability that a subject would experience an adverse or catastrophic landing. However, use of motion parallax was not a significant predictor of either adverse or catastrophic landings in any model (Table 1). The insignificant effect of motion parallax use on landing outcome may derive from the animals' use of this cue only in the initial stages of a leap. Motion parallax is likely an impractical cue to use mid-leap to prepare for landing given the time required to generate the head movements necessary for motion parallax cues, and subjects were never observed making parallax motions mid-leap.

The insignificant effect on performance of a depth cue used exclusively during launching highlights the importance of depth cues for coordinating precise landings at the end of a leap in addition to their role in appropriately calibrating launch power and direction during take-off. Subjects significantly altered their launching behavior under the reduced binocular field condition by changing their horizontal speed, path choice and motion parallax use (but not take-off lag; Table 2). However, even when the effects of these adjustments were controlled statistically, subjects exhibited decreased landing performance under the reduced binocular field condition as indicated by their increased probability of experiencing adverse landings and requiring additional grasp adjustments. This importance of binocular visual cue availability for subjects' ability to execute precise landings runs counter to the suggestion that leaping behavior could not have selected for more forward-facing eyes in primates because landing substrates were outside of the functional range of stereopsis at the start of a leap (Heesy, 2009). Instead, the effect of binocular cue availability on landing precision regardless of launch adjustments suggests that even if early primates were engaging in leaps long enough that the range of stereopsis at take-off would not include the landing substrate, stereopsis could still have been important in facilitating preparation for a coordinated landing while the animal is in the air. These results are also congruent with disruption of optic flow cues about speed and heading of movement. Assuming an animal held its head with the snout pointed in the direction of travel, the blinder would have at least partially blocked the portion of the monocular visual field of the occluded eye within which the center of expansion would fall, potentially reducing the animal's access to optic flow cues about the speed at which they are approaching the landing substrate. Optic flow cues, like stereoscopic cues and in contrast to motion parallax cues, could also potentially be used throughout the course of the leap.

**Table 2.**
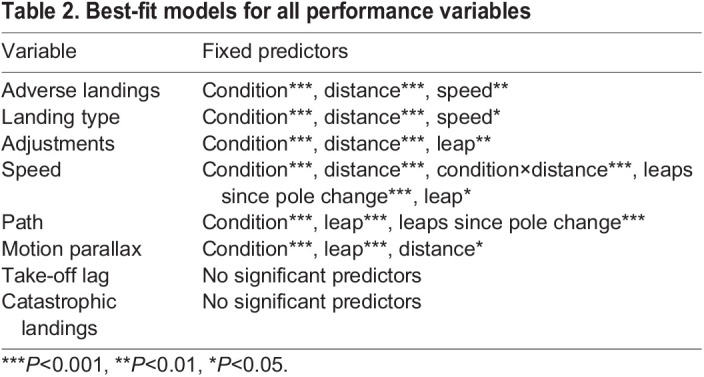
Best-fit models for all performance variables

Greater availability of binocular visual cues allowed the subjects in this study to leap further, faster and with more precisely grasped landings. While it is not possible to determine whether these differences are attributable to the stereoscopic depth cues, potentially improved optic flow cues or sensitivity improvements that are created within the binocular field, these results suggest that the overall impact of increased availability of binocular visual cues on grasp-leaping locomotion is improved efficiency, potentially in terms of both speed and energetic expenditure. How early crown primates used leaping locomotion would determine exactly how improved grasp-leaping efficiency due to a wider binocular field may have affected the ecology of the earliest crown primates. Suggestions for the biological role of leaping in early primates have ranged from a more efficient way to navigate a complex and discontinuous arboreal habitat (Szalay and Dagosto, 1980; Szalay, 2007), to a mechanism of predator avoidance (Crompton and Sellers, 2007), to a method of ambushing insect prey (Boyer et al., 2016). Performance of behaviors that influence locomotor efficiency, predator avoidance, prey capture, or any combination of the three are likely to make substantial contributions to an individual's fitness and thus be under intense selection. Therefore, the improvement in grasp-leaping efficiency afforded by more forward-facing eyes would likely have been a target for strong selection regardless of which ecological role leaping predominantly filled in the earliest primates.

Phylogenetic and functional studies of primate and euarchontan tarsal evolution suggest that moderate leaping adaptations were present in the earliest crown primates (Boyer et al., 2013a,b; Marigó et al., 2016; Boyer et al., 2017; but see Dunn et al., 2016). The tarsal anatomy of the dentally primitive *Donrusellia provincialis* as well as reconstructions of adaptive shifts in the functional morphology of the hand and foot across Euarchonta have been used to suggest that leaping adaptations may have even preceded morphological commitment to the fine branch niche in early primates (Boyer and Seiffert, 2013; Boyer et al., 2013b, 2016, 2017; Yapuncich et al., 2017). These findings suggest a persistent selective pressure for improved performance in acrobatic leaping both in the earliest crown primates and in the strepsirrhine and haplorhine stem lineages, which would have favored features that improved leaping performance. The results of this experiment indicate that increased availability of binocular visual cues significantly improves leaping performance in a nocturnal small-bodied generalized arboreal quadrupedal taxon with facultative leaping. While similar experiments in *C. medius* and *Microcebus murinus* found that increased binocular cue availability provides a more substantial improvement in insect predation success (performance improvements >23%, Kemp, 2024), suggesting that the demands of capturing mobile prey were likely the primary driver of the evolution of primates' forward-facing eyes, this study presents a functional mechanism by which selection for improved grasp-leaping could also have contributed to selection for forward-facing eyes in the earliest crown primates.

## Supplementary Material

10.1242/jexbio.245434_sup1 Supplementary information

## References

[JEB245434C1] Bates, D., Mächler, M., Bolker, B. and Walker, S. (2015). Fitting linear mixed-effects models using lme4. *J. Statistical. Software* 67, 1-48. 10.18637/jss.v067.i01

[JEB245434C2] Bloch, J. I. and Boyer, D. M. (2002). Grasping primate origins. *Science* 298, 1606-1610. 10.1126/science.1078249 12446906

[JEB245434C3] Bloch, J. I. and Silcox, M. T. (2006). Cranial anatomy of the Paleocene plesiadapiform *Carpolestes simpsoni* (Mammalia, Primates) using ultra high-resolution X-ray computed tomography, and the relationships of plesiadapiforms to Euprimates. *J. Hum. Evol.* 50, 1-35. 10.1016/j.jhevol.2005.06.009 16236344

[JEB245434C4] Bloch, J. I., Silcox, M. T., Boyer, D. M. and Sargis, E. J. (2007). New Paleocene skeletons and the relationship of plesiadapiforms to crown-clade primates. *Proc. Natl. Acad. Sci. USA* 104, 1159-1164. 10.1073/pnas.0610579104 17229835 PMC1783133

[JEB245434C5] Boyer, D. M. and Seiffert, E. R. (2013). Patterns of astragalar fibular facet orientation in extant and fossil primates and their evolutionary implications. *Am. J.Phys. Anthrop.* 151, 420-447. 10.1002/ajpa.22283 23794333

[JEB245434C6] Boyer, D. M., Seiffert, E. R., Gladman, J. T. and Bloch, J. I. (2013a). Evolution and allometry of calcaneal elongation in living and extinct primates. *PLoS One* 8, e67792. 10.1371/journal.pone.0067792 23844094 PMC3701013

[JEB245434C7] Boyer, D. M., Yapuncich, G. S., Chester, S. G. B., Bloch, J. I. and Godinot, M. (2013b). Hands of early primates. *Am. J.Phys. Anthropol.* 152, 33-78. 10.1002/ajpa.22392 24249591

[JEB245434C8] Boyer, D. M., Yapuncich, G. S., Chester, S. G. B., Bloch, J. I. and Godinot, M. (2016). Hands of paleogene primates. In *The Evolution of the Primate Hand, Developments in Primatology: Progress and Prospects* (ed. T. L. Kivell, P. Lemelin, B. G. Richmond and D. Schmitt), pp. 373-419. New York, NY: Springer New York.

[JEB245434C9] Boyer, D. M., Toussaint, S. and Godinot, M. (2017). Postcrania of the most primitive euprimate and implications for primate origins. *J. Hum. Evol.* 111, 202-215. 10.1016/j.jhevol.2017.07.005 28874272

[JEB245434C10] Campbell, F. W. and Green, D. G. (1965). Monocular versus binocular visual acuity. *Nature* 208, 191-192. 10.1038/208191a0 5884255

[JEB245434C11] Cartmill, M. (1972). Arboreal adaptations and the origin of the order Primates. In *The Functional and Evolutionary Biology of Primates* (ed. R. Tuttle), pp. 97-122. Aldine, Chicago.

[JEB245434C53] Cartmill, M. (1974). Rethinking primate origins: the characteristic primate traits cannot be explained simply as adaptations to arboreal life. *Science* 184, 436-443. 4819676 10.1126/science.184.4135.436

[JEB245434C12] Cartmill, M. (1992). New views on primate origins. *Evol. Anthropol* .1, 105-111. 10.1002/evan.1360010308 23280918

[JEB245434C13] Crompton, R. H. (1995). “Visual Predation”, habitat structure, and the ancestral primate niche. In *Creatures of the Dark* (ed. L. Alterman), pp. 11-30. Springer.

[JEB245434C14] Crompton, R. H. and Sellers, W. I. (2007). A consideration of leaping locomotion as a means of predator avoidance in prosimian primates. In *Primate Anti-Predator Strategies* (ed. S. Gursky-Doyen and K. A. I. Nekaris), pp. 127-145. Boston, MA: Springer.

[JEB245434C15] Dunn, R. H., Rose, K. D., Rana, R. S., Kumar, K., Sahni, A. and Smith, T. (2016). New euprimate postcrania from the early Eocene of Gujarat, India, and the strepsirrhine–haplorhine divergence. *J. Hum. Evol.* 99, 25-51. 10.1016/j.jhevol.2016.06.006 27650579

[JEB245434C16] Gebo, D. L., Smith, T. and Dagosto, M. (2012). New postcranial elements for the earliest Eocene fossil primate *Teilhardina belgica*. *J. Hum. Evol.* 63, 205-218. 10.1016/j.jhevol.2012.03.010 22704262

[JEB245434C17] Gebo, D. L., Smith, R., Dagosto, M. and Smith, T. (2015). Additional postcranial elements of Teilhardina belgica: The oldest European primate. *Am. J. Phys. Anthropol.* 156, 388-406. 10.1002/ajpa.22664 25388600

[JEB245434C18] Gray, R. and Regan, D. (1998). Accuracy of estimating time to collision using binocular and monocular information. *Vis. Res.* 38, 499-512. 10.1016/S0042-6989(97)00230-7 9536374

[JEB245434C54] Heesy, C. P. (2004). On the relationship between orbit orientation and binocular visual field overlap in mammals. *Anat. Rec. A* 1, 1104-1110. 10.1002/ar.a.20116 15470671

[JEB245434C19] Heesy, C. P. (2009). Seeing in stereo: The ecology and evolution of primate binocular vision and stereopsis. *Evol. Anthropol.* 18, 21-35. 10.1002/evan.20195

[JEB245434C20] Howard, I. P. and Rogers, B. J. (1995). *Binocular Vision and Stereopsis*. New York: Oxford University Press.

[JEB245434C21] Kandel, E. R. (1991). Perception of motion, depth, and form. In *Principles of Neural Science* (ed. E. R. Kandel, J. H. Schwartz and T. M. Jessell), pp. 440-466. Appleton & Lange.

[JEB245434C55] Kemp A. D. (2019). Adaptive significance of primate binocular vision in grasping and locomotion. PhD thesis, University of Texas at Austin.

[JEB245434C56] Kemp, A. D. (2024). Effect of binocular visual cue availability on fruit and insect grasping performance in two cheirogaleids: Implications for primate origins hypotheses. *J. Hum. Evol.* 188, 103456. 10.1016/j.jhevol.2023.103456 38325119

[JEB245434C22] Kirk, E. C., Cartmill, M., Kay, R. F. and Lemelin, P. (2003). Comment on “Grasping Primate Origins.”. *Science* 300, 741. 10.1126/science.1081587 12730582

[JEB245434C23] Kirk, E. C., Lemelin, P., Hamrick, M. W., Boyer, D. M. and Bloch, J. I. (2008). Intrinsic hand proportions of euarchontans and other mammals: Implications for the locomotor behavior of plesiadapiforms. *J. Hum. Evol.* 55, 278-299. 10.1016/j.jhevol.2008.02.008 18440594

[JEB245434C57] Lee D. N. (1980). The optic flow field: The foundation of vision. *Phil. Trans. R. Soc. Lond. B* 290, 169-179. 6106236 10.1098/rstb.1980.0089

[JEB245434C24] Lee, D. N. and Reddish, P. E. (1981). Plummeting gannets: a paradigm of ecological optics. *Nature* 293, 293-294. 10.1038/293293a0

[JEB245434C25] Lenth, R. V. (2016). Least-squares means: the r package lsmeans. *J. Stat. Softw.* 69, 1-33. 10.18637/jss.v069.i01

[JEB245434C26] Marigó, J., Roig, I., Seiffert, E. R., Moyà-Solà, S. and Boyer, D. M. (2016). Astragalar and calcaneal morphology of the middle Eocene primate *Anchomomys frontanyensis* (Anchomomyini): Implications for early primate evolution. *J. Hum. Evol.* 91, 122-143. 10.1016/j.jhevol.2015.08.011 26852816

[JEB245434C27] Martin, R. D. (1979). Phylogenetic aspects of prosimian behavior. In *The Study of Prosimian Behavior* (ed. G. A. Doyle), pp. 45-77. New York: Academic Press.

[JEB245434C28] Moore, D. S. and McCabe, G. P. (1999). *Introduction to the Practice of Statistics*. New York: W. H. Freeman.

[JEB245434C29] Ni, X., Wang, Y., Hu, Y. and Li, C. (2004). A euprimate skull from the early Eocene of China. *Nature* 417, 65-67. 10.1038/nature02126 14702085

[JEB245434C30] Pettigrew, J. D. (1986). The evolution of binocular vision. In *Visual Neuroscience* (ed. J. D. Pettigrew, K. J. Sanderson and W. R. Levick), pp. 208-222. London: Cambridge University Press.

[JEB245434C31] Pirenne, M. H. (1943). Binocular and uniocular threshold of vision. *Nature* 152, 698-699. 10.1038/152698a0

[JEB245434C32] Plooy, A., Tresilian, J. R., Mon-Williams, M. and Wann, J. P. (1998). The contribution of vision and proprioception to judgements of finger proximity. *Exp. Brain Res.* 118, 415-420. 10.1007/s002210050295 9497148

[JEB245434C33] Poggio, G. F. and Poggio, T. (1984). The analysis of stereopsis. *Annu. Rev. Neurosci.* 7, 379-412. 10.1146/annurev.ne.07.030184.002115 6370081

[JEB245434C34] Purves, D., Augustine, G. J., Fitzpatrick, D., Hall, W., LaMantia, A. S., McNamara, J. O. and Williams, S. M. (2012). *Neuroscience*, 4 edn. Sinauer Associates Incorporated.

[JEB245434C35] Ravosa, M. J. and Savakova, D. G. (2004). Euprimate origins: The eyes have it. *J. Hum. Evol.* 46, 357-364. 10.1016/j.jhevol.2003.12.002 14984789

[JEB245434C36] Read, J. C. (2021). Binocular vision and stereopsis across the animal kingdom. *Annual Review Vis. Sci.* 7, 389-415. 10.1146/annurev-vision-093019-113212 34283925

[JEB245434C37] Rock, I. (1984). *Perception*. New York: Scientific American Library.

[JEB245434C38] Ross, C. F. (2000). Into the Light: The Origin of Anthropoidea. *Annu. Rev. Anthropol*. 29, 147-194. 10.1146/annurev.anthro.29.1.147

[JEB245434C39] Ross, C. F., Hall, M. I. and Heesy, C. P. (2007). Were basal primates nocturnal? evidence from eye and orbit shape. In *Primate Origins: Adaptations and Evolution* (ed. M. J. Ravosa and M. Dagosto), pp. 233-256. Boston, MA: Springer US.

[JEB245434C40] Schielzeth, H., Dingemanse, N. J., Nakagawa, S., Westneat, D. F., Allegue, H., Teplitsky, C., Réale, D., Dochtermann, N. A., Garamszegi, L. Z. and Araya-Ajoy, Y. G. (2020). Robustness of linear mixed–effects models to violations of distributional assumptions. *Methods Ecol. Evol.* 11, 1141-1152. 10.1111/2041-210X.13434

[JEB245434C41] Schwarz, G. (1978). Estimating the dimension of a model. *Annals Stat.* 6, 461-464. 10.1214/aos/1176344136

[JEB245434C42] Silcox, M. T., Sargis, E. J., Bloch, J. I. and Boyer, D. M. (2014). Morphological evidence for primate origins and supraordinal relationships. *Handbook Paleoanthropol* 1, 1-27.

[JEB245434C43] Sussman, R. W. (1995). How primates invented the rainforest and vice versa. In *Creatures of the Dark* (ed. L. Alterman, G. A. Doyle and M. K. Izard). Springer. 10.1007/978-1-4757-2405-9_1

[JEB245434C44] Sussman, R. W. and Raven, P. H. (1978). Primate origins and the evolution of angiosperms. *A. J. Primatol.* 23, 209-223. 10.1002/ajp.1350230402 31952400

[JEB245434C45] Sussman, R. W., Rasmussen, D. T. and Raven, P. H. (2013). Rethinking primate origins again. *A. J. Primatol.* 75, 95-106. 10.1002/ajp.22096 23184701

[JEB245434C46] Szalay, F. S. (2007). Ancestral locomotor modes, placental mammals and the origin of eurprimates: Lessons from history. In *Primate Origins: Adaptation and Evolution* (ed. M. L. Ravosa and M. Dagosto), pp. 457-487. Boston, MA: Springer US.

[JEB245434C47] Szalay, F. S. and Dagosto, M. (1980). Locomotor adaptations as reflected in the adaptations of Paleogene primates. *Fol. Primatol.* 34, 1-45. 10.1159/000155946 7002751

[JEB245434C48] Szalay, F. S. and Dagosto, M. (1988). Evolution of hallucial grasping in primates. *J. Hum. Evol.* 17, 1-33. 10.1016/0047-2484(88)90047-4

[JEB245434C49] Warren, W. H. Jr, Kay, B. A., Zosh, W. D., Duchon, A. P. and Sahuc, S. (2001). Optic flow is used to control human walking. *Nature Neurosci.* 4, 213-216. 10.1038/84054 11175884

[JEB245434C50] Warrant, E. (2008). Nocturnal vision. In *The Senses: a Comprehensive Reference*, Vol. 2, Vision II (ed. A. I. Basbaum, A. Kaneko, G. M. Shepherd and G. Westheimer), pp. 53-86. Academic Press.

[JEB245434C51] Wickham, H. (2009). *ggplot2: Elegant Graphics for Data Analysis*. New York: Springer-Verlag.

[JEB245434C52] Yapuncich, G. S., Seiffert, E. R. and Boyer, D. M. (2017). Quantification of the position and depth of the flexor hallucis longus groove in euarchontans, with implications for the evolution of primate positional behavior. *Am. J. Phys. Anthropol.* 83, 373. 10.1002/ajpa.23213 28345775

