# Supplementary material for: Effects of binocular cue availability on leaping performance in Cheirogaleus medius: implications for primate origins
Source: J Exp Biol. 2024 Feb 22;227(4):jeb245434. doi: 10.1242/jeb.245434 (PMC10918687; doi:10.1242/jeb.245434)
Supplement: Supplementary information [file jexbio-227-245434-s1.pdf]

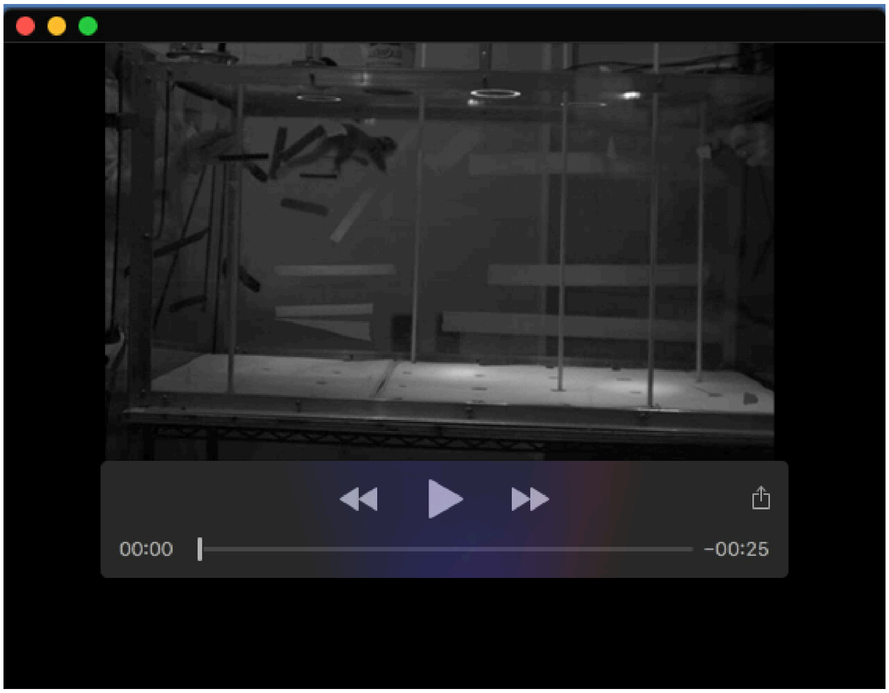

Movie 1. An example of a *spin*, an adverse landing in which the subject is not able to counter the rotational momentum it creates upon landing and rotates at least 90 around the substrate.

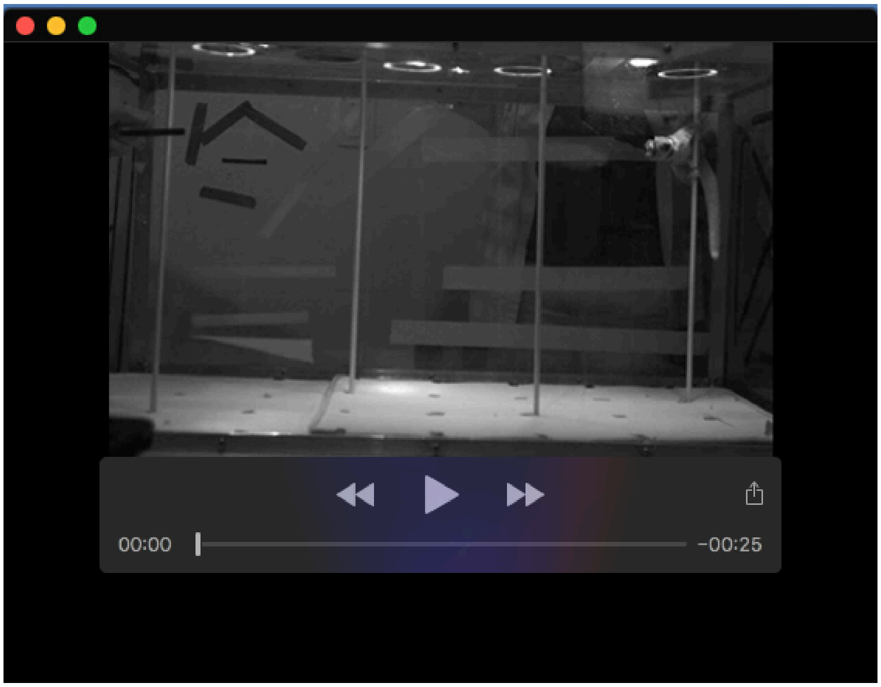

Movie 2. An example of a *strike*, an adverse landing in which the subject's face or torso contacts the landing substrate before their hands or feet.

Journal of Experimental Biology • Supplementary information

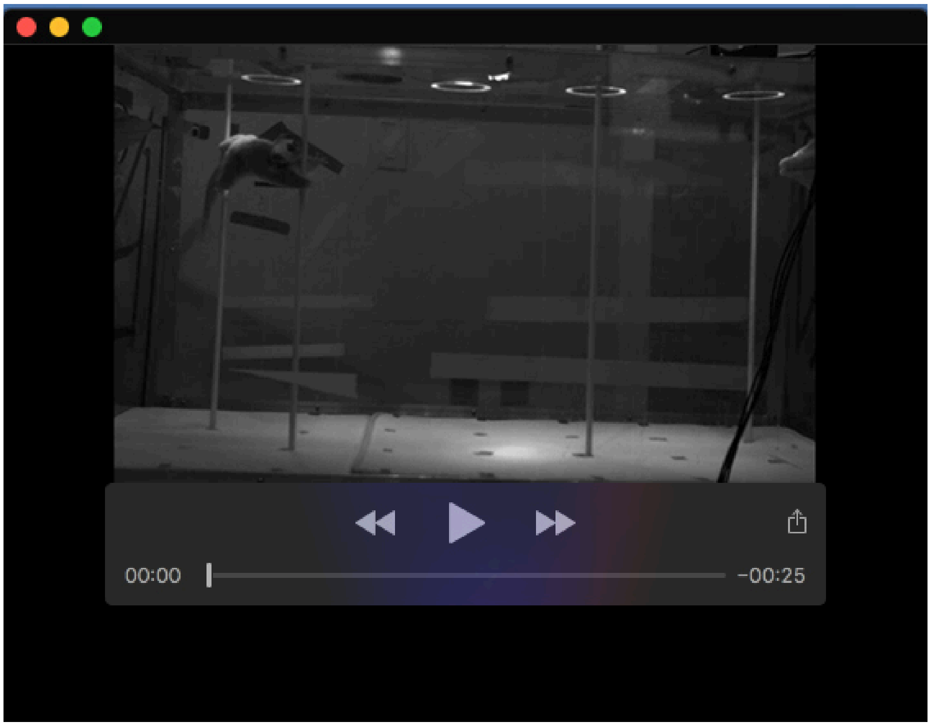

Movie 3. An example of a *drop*, an adverse landing in which the subject fails to get a secure grip on the substrate and falls at least 5 cm before managing to arrest themselves. In this example, the drop is preceded by a strike.

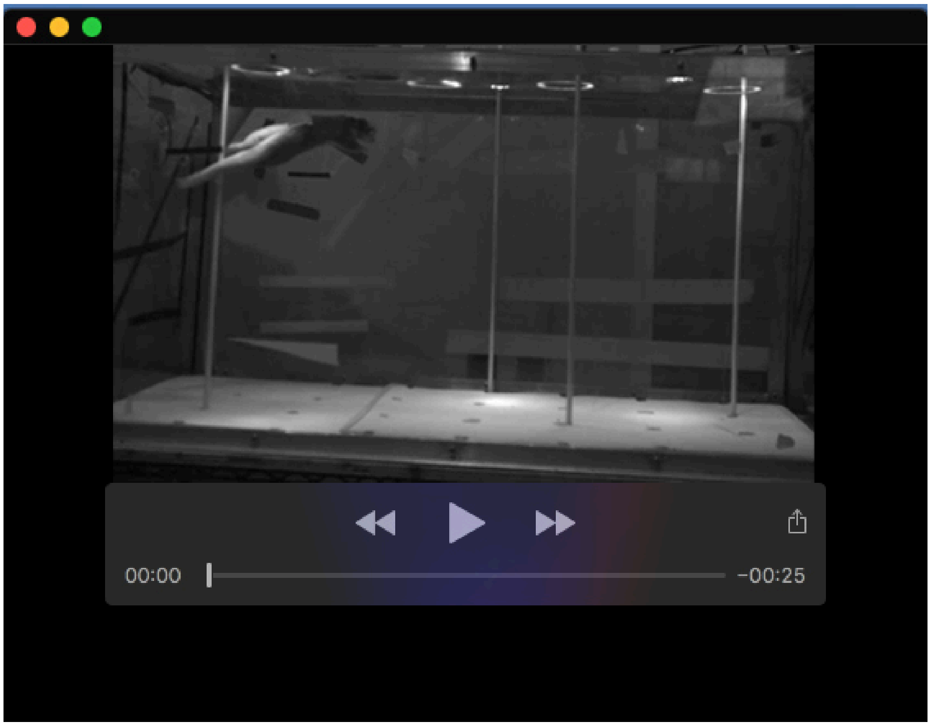

Movie 4. An example of a *fall*, an adverse landing in which the subject completely fails to contact the vertical substrate and lands on the bottom of the enclosure.
